# Data on importance of hematopoietic cell derived Lipocalin 2 against gut inflammation

**DOI:** 10.1016/j.dib.2016.06.047

**Published:** 2016-07-02

**Authors:** Piu Saha, Vishal Singh, Xia Xiao, Beng San Yeoh, Matam Vijay-Kumar

**Affiliations:** a Department of Nutritional Sciences, The Pennsylvania State University, University Park, PA 16802, United States; b Department of Medicine, The Pennsylvania State University Medical Center, Hershey, PA 17033, United States

**Keywords:** Siderocalin, Gut microbiota, Inflammatory bowel disease, Bone marrow chimeras, Colitis

## Abstract

The data herein is related to the research article entitled “Microbiota-inducible innate immune siderophore binding protein Lipocalin 2 is critical for intestinal homeostasis” (Singh et al., 2016) [1]. In the present article, we monitored dextran sodium sulfate (DSS)-induced colitis development upon Lipocalin 2 (Lcn2) neutralization, and examined the survival of Lcn2 deficient (Lcn2KO) mice and their WT littermates upon DSS challenge. To dissect the relative contribution of immune and non-immune cells-derived Lcn2 in mediating protection against gut inflammation, we generated respective bone marrow chimera and evaluated their susceptibility to IL-10 receptor neutralization-induced chronic colitis.

Neutralization of Lcn2 in WT mice resulted in exacerbated DSS-induced colitis. Notably, mice lacking Lcn2 exhibited 100% mortality whereas only 20% mortality was observed in WT mice upon DSS challenge. Further, data from bone marrow chimera showed that immune cell-derived Lcn2 is the major contributor in conferring protection against colitis.

## Specification Table

**Table:** 

Subject area	Biology
More specific subject area	Lipocalin 2, inflammatory bowel disease
Type of data	Graphs, figures
How data was acquired	Assessment of colitis parameters, percent survival, myeloperoxidase assay, enzyme linked immunosorbent assay (ELISA), Biotek Eon™ microplate spectrophotometer.
Data format	Analyzed
Experimental factors	The susceptibility to DSS-induced colitis was assessed in Lcn2KO mice and their WT littermates. Lcn2 bone marrow chimeras was generated and evaluated for susceptibility to IL-10 receptor neutralization-induced chronic colitis.
Experimental features	Percent survival and analysis of standard colitis parameters
Data source location	Pennsylvania, USA
Data accessibility	Data are provided with this article

## Value of the data

• The data are valuable to researchers interested in investigating the role of Lcn2 in inflammatory bowel disease.
• The data provide information on protective roles of immune vs non-immune cell derived Lcn2 during gut inflammation.
• The data support future studies in delineating the role of Lcn2 in mucoprotection.

## 1 Data

**Fig. 1:**
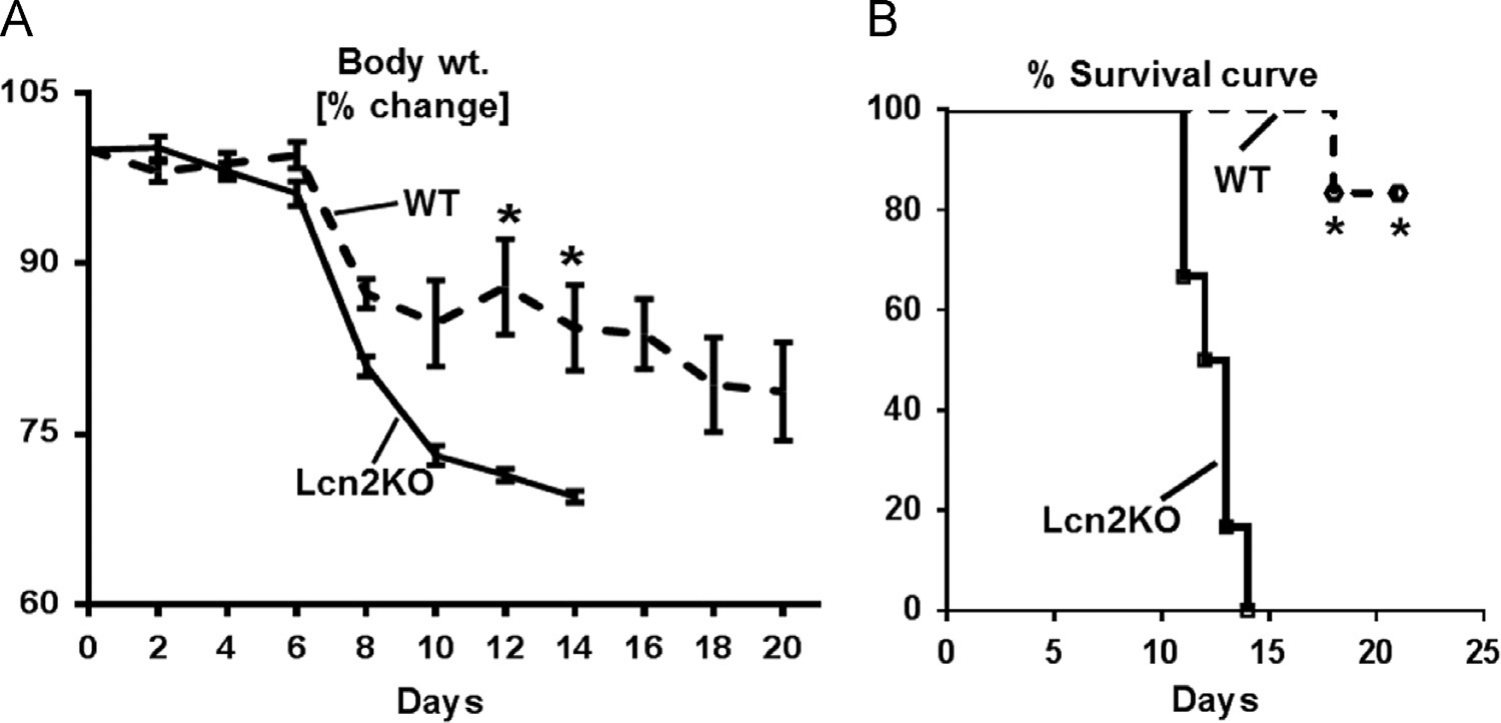
Data on the susceptibility of Lcn2KO mice to DSS-induced acute colitis**.** (A) Percent body weight loss of DSS-treated Lcn2KO mice and their WT littermates (*n*=5). (B) Percent survival of WT and Lcn2KO mice upon challenge with 1.5% DSS for 7 days (*n*=5). Data are expressed as mean±SEM. One-way analysis of variance with the Tukey multiple comparison test were used. *p<0.05 was considered statistically significant.

**Fig. 2:**
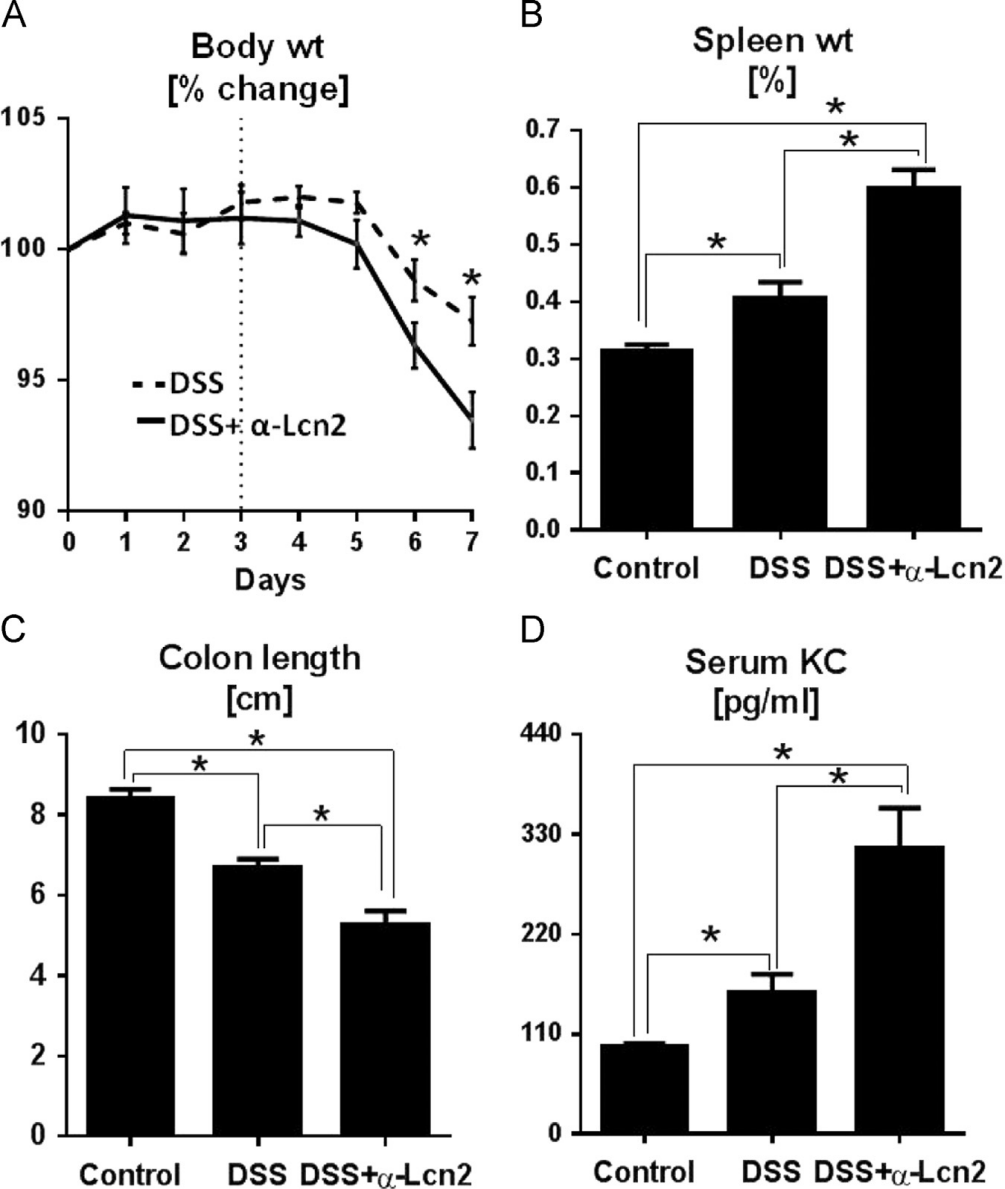
Data on effect of Lcn2-neutralization on severity of colitis. WT mice (female, 8 weeks old, *n*=5) were administered 1.5% DSS for 7 days. On day 3 onwards up to day 7, colitic mice were administered once/day with either anti-mouse Lcn2 mAb (mLcn2, 20 µg/mouse/day, intraperitoneally) or isotype antibody (IgG1). After 7 days, mice were euthanized and analyzed for standard colitis parameters. (A) Body weight, (B) spleen weight, (C) colon thickening and (D) serum KC. Data are expressed as mean±SEM. Unpaired *t*-test was used to compare the difference between groups. *p<0.05 was considered statistically significant.

**Fig. 3:**
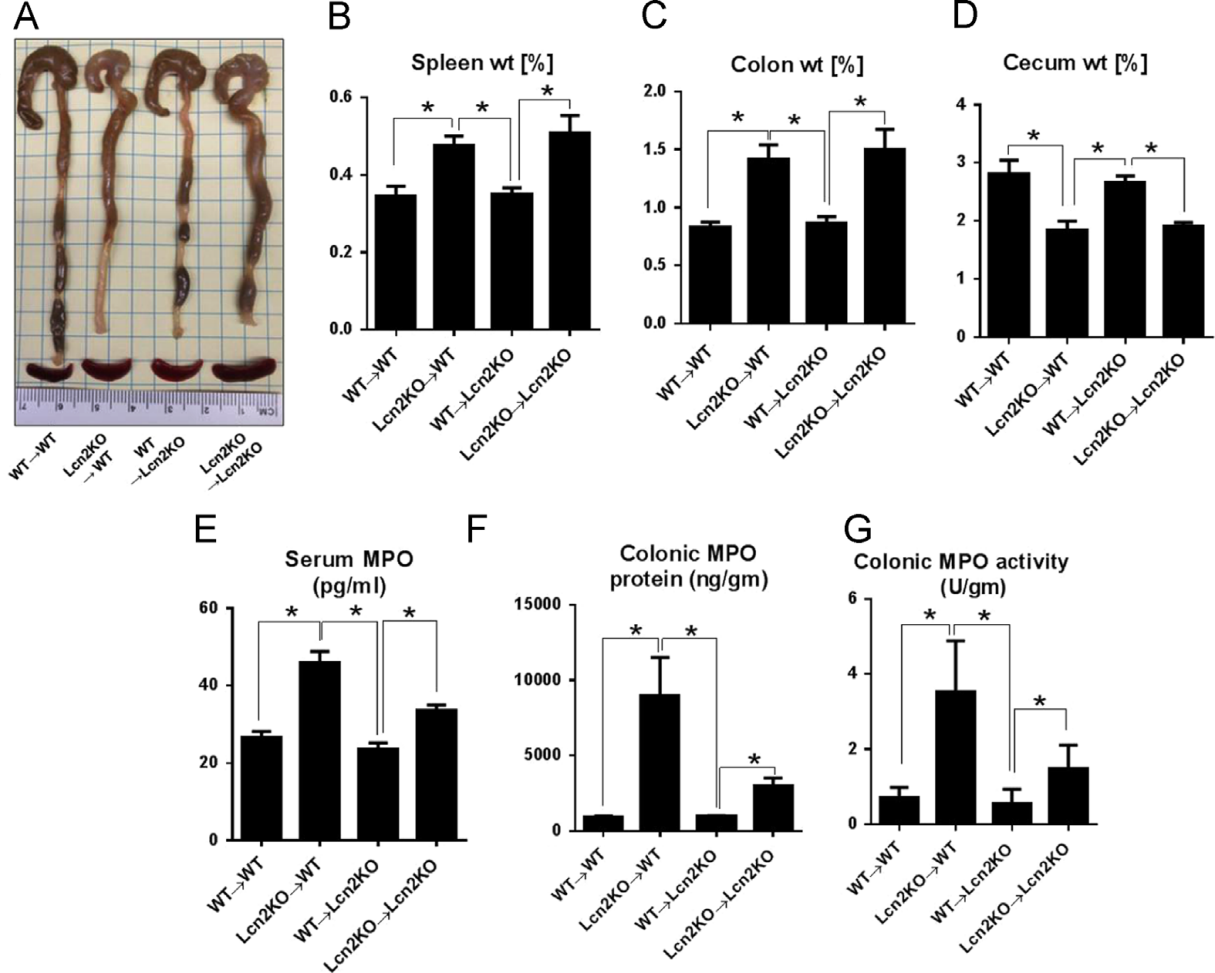
Contribution of immune and non-immune cells derived Lcn2 on the severity of colitis in Lcn2 bone marrow chimeric mice. To explore the relative contribution of immune and non-immune cells-derived Lcn2 in severity of colitis, Lcn2 bone marrow chimeric mice were treated with IL-10R-neutralizing antibody (1.0 mg/mouse, intraperitoneally, once/week) for 4 weeks (*n*=4). One week after the fourth injection, mice were euthanized and analyzed for standard colitis parameters. (A) Gross picture of colon and spleen, (B) spleen weight, (C) colon weight, (D) cecum weight, (E) serum MPO, (F) colonic MPO protein level and (G) colonic MPO activity. The constitution of the bone marrow chimeric mice is denoted as (BM donors) – (recipient mice) accordingly. Data are expressed as mean±SEM. Unpaired *t*-test was used. *p<0.05 was considered statistically significant.

In the data, we presented the higher mortality rate of Lcn2KO mice in DSS-induced acute colitis when compared to their WT littermates (Fig. 1). Further, neutralization of Lcn2 in DSS-treated WT mice resulted in exacerbated gut inflammation as indicated by greater body weight loss, splenomegaly, colon thickening, and serum KC (Fig. 2). Experiments with bone marrow chimeric mice revealed that immune cells-derived Lcn2 are the major contributor in mediating protection against IL-10R neutralization-induced chronic colitis (Fig. 3) indicated by reduction in splenomegaly, colomegaly, shrunken ceca, MPO protein levels and activity.

## 2 Experimental design, materials and methods

### 2.1 DSS induced mortality

Eight weeks old female C57BL/6 WT and Lcn2KO mice (*n*=5) were administered 1.5% DSS (MP Biomedicals Inc.) in drinking water for 7 days. Colonic inflammation was examined by fecal occult blood, diarrhea, and loss of body weight. After 7 days, mice were switched to regular drinking and subsequently monitored for mortality up to 21 days.

### 2.2 Lipocalin 2 (Lcn2) neutralization in DSS-induced colitis model

Female WT mice (8 weeks old; *n*=5) were administered 1.5% DSS in drinking water for 7 days. On day 3 onwards up to day 7, mice were administered once daily with anti-mouse Lcn2 monoclonal antibody (20 µg/mouse/day, intraperitoneally; R&D Systems). Control mice received isotype (IgG1) control. On day 7, mice were euthanized by CO_2_ asphyxiation and blood samples were collected in a serum separating tube (Becton Dickinson). Upon centrifugation, the hemolysis-free serum were collected and stored at −80 °C until further analysis. Standard colitis parameters (body weight, spleen weight, occult blood and colon shortening) were assessed. Serum KC were analyzed by ELISA according to the manufacturer׳s protocol [2].

### 2.3 Bone marrow chimera generation and neutralization of IL-10R

Eight weeks old Lcn2KO mice and their WT littermates were employed to generate WT→WT, WT→Lcn2KO, Lcn2KO→WT, and Lcn2KO→Lcn2KO bone marrow chimeras (BMC; denoted as bone marrow donor→recipients) as described previously [1]. 95–99% chimerism was confirmed by determining the differential CD45.1 and CD45.2 expression in PBMC of recipient mice via flow cytometry [3], [4].

After 8 weeks post-transplant, Lcn2 bone marrow chimeric mice (*n*=4) were administered anti-mouse α-IL10R mAb (1.0 mg/mouse, intraperitoneally; BioXcell) once weekly for total of 4 weeks to induce chronic colitis as described earlier [1]. Control mice received isotype (IgG1) control. Mice were regularly monitored for fecal occult blood and change in body weight. One week after the fourth injection, the mice were euthanized and analyzed for standard colitis parameters [5]. MPO levels in the serum and colon lysate were measured by ELISA according to the manufacturer׳s protocol.

### 2.4 Statistical analysis

Data are expressed as mean±SEM. The significance of difference between two groups was determined by unpaired student *t*-test. The statistical significance between 3 or more groups were analyzed by one-way ANOVA with Tukey׳s post-hoc test. *p* value less than 0.05 were considered statistically significant. Statistical significance was calculated using Graph Pad Prism version 6.0 (Graph Pad Software Inc.).
