# Supplementary material for: Data on importance of hematopoietic cell derived Lipocalin 2 against gut inflammation
Source: Data Brief. 2016 Jul 2;8:812–6. doi: 10.1016/j.dib.2016.06.047 (PMC4956906; doi:10.1016/j.dib.2016.06.047)
Supplement: Supplementary file 1 — Supplementary material [file mmc1.docx]

The authors have no conflict of interest.
